# New T2T assembly of *Cryptosporidium parvum* IOWA II annotated with Legacy-Compatible Gene identifiers

**DOI:** 10.1038/s41597-025-05364-3

**Published:** 2025-06-19

**Authors:** Rodrigo de Paula Baptista, Rui Xiao, Yiran Li, Travis C. Glenn, Jessica C. Kissinger

**Affiliations:** 1 Center for Tropical and Emerging Global Diseases, University of Georgia, Athens, GA 30602 USA; 2 Institute of Bioinformatics, University of Georgia, Athens, GA 30602 USA; 3 Environmental Health Science, University of Georgia, Athens, GA 30602 USA; 4 Department of Genetics, University of Georgia, Athens, GA 30602 USA; 5 Present Address: Houston Methodist Research Institute, Houston, TX 77030 USA; 6 Present Address: St. Jude Children’s Research Hospital, Memphis, TN 38105 USA

**Keywords:** Genome, Parasite genomics

## Abstract

*Cryptosporidium parvum* is a significant pathogen causing gastrointestinal infections in humans and animals. It is spread through ingesting contaminated food and water. Despite its global health significance, generating a *C. parvum* genome sequence has been challenging for many reasons including cloning and challenging subtelomeric regions. A new, gapless, hybrid, telomere-to-telomere genome assembly was created for *C. parvum* IOWA II, here termed *Cp*BGF. It reveals 8 chromosomes, a genome size of 9,259,183 bp, and resolves complex subtelomeric regions. To facilitate ease of use and consistency with the literature, the chromosomes have been oriented, and genes in this annotation have been given similar gene IDs as those used in the 2004, *C. parvum* IOWA II reference genome sequence. The new annotation utilized considerable RNA expression evidence including single-molecule Iso-Seq data; thus, untranslated regions, long noncoding RNAs, and antisense RNAs are annotated. The *Cp*BGF genome assembly serves as a valuable resource for understanding the biology, pathogenesis, and transmission of *C. parvum*, and it facilitates the development of diagnostics, drugs, and vaccines against cryptosporidiosis.

## Background & Summary

*Cryptosporidium parvum* is an apicomplexan parasite that infects the gastrointestinal tract of humans and animals. It is a leading cause of waterborne disease outbreaks worldwide and is responsible for significant morbidity and mortality in immunocompromised individuals and infants^1^. *Cryptosporidium* infection occurs through ingesting contaminated water, food, or fecal matter, making it a significant public health concern^2^.

Cryptosporidiosis is a neglected disease that has historically been underdiagnosed and underreported^3,4^. Although improvements in diagnostic techniques have led to increased detection and reporting of cases, the disease burden is likely underestimated due to a lack of surveillance in many countries. The disease is particularly concerning in developing countries, where access to clean water and sanitation is limited, and outbreaks are common. In industrialized nations, including the U.S., cryptosporidiosis is one of the major waterborne diseases related to diarrhea and death in children and immunocompromised patients^5^.

Despite the importance of *Cryptosporidium* as a human and animal pathogen, sequencing its genome has been challenging due to its repetitive subtelomeric ends and difficulty in obtaining enough material for DNA extraction because of the lack of sufficient *in vitro* cultivation systems to grow enough of these parasites^6^. Currently, at NCBI, >50 whole genome assemblies are available for the genus, representing ~ 15 species^7^. However, only the *Cryptosporidium parvum* IOWA II genome sequence has chromosomal physical mapping information available^8^. Due to the challenges of obtaining material and the extensive use of short-read sequencing, most assemblies are fragmented and incomplete, limiting the community’s ability to easily compare genome sequences and link them to the parasite’s biology, virulence mechanisms, and transmission dynamics^9^.

Recent advances in sequencing technologies have enabled the production of high-quality *Cryptosporidium* genome assemblies, which can provide an almost complete, if not full representation of the genome sequence without physical mapping information^10,11^. However, some regions of the *Cryptosporidium* genome sequence are still challenging, including the subtelomeric regions of chromosomes 1, 7, and 8, which appear to have been replicated among these three chromosomes and also contain important multi-copy genes such as copies of 18S and 28S rRNA genes, tryptophan synthase beta and the MEDLE genes^10^. With deep sequencing and a combination of recent bioinformatics approaches, we present the first telomere-to-telomere genome assembly of *C. parvum* IOWA II obtained from Bunch Grass Farms (*Cp*BGF), which includes placement of all subtelomeric regions and telomeres. Importantly, this new genome assembly mirrors the majority of the current reference genome assembly^8^, including chromosomal orientation and gene naming, to facilitate comparisons with the reference genome and existing literature. The new assembly has also resolved the unresolved regions in chromosomes 2, 4, and 5 due to a lack of physical mapping to support scaffolding found in the original, *C. parvum* IOWA II strain (*Cp*IOWA II) reference assembly^8^. The new *Cp*BGF assembly resolves all 16 subtelomeric regions and telomeres, and the annotation contains new ncRNA information, including lncRNAs^12^ and sncRNAs^13^, and UTR boundaries and transcript isoforms have been added based on RNA long-read data. Although chromosomal-level genome assemblies such as *Cp*IOWA-ATCC and *Cp*BCM-BGF are nearly complete, they lack the chromosomal orientation established in the original reference genome and remain incomplete in certain subtelomeric regions. Furthermore, each assembly’s gene annotations utilize unique gene identifiers, which hinders cross-study comparisons and limits their utility as a standardized reference within the research community. Given the widespread use of the *Cp*IOWA II BGF isolates due to their commercial availability, having a *Cp*IOWA II from BGF as a reference should facilitate research. Statistics of how the new assembly and annotation compare to previous assemblies and annotation are located below in the Technical Validation section.

*C. parvum* IOWA II is among the most commonly used *C. parvum* strains in the laboratory. The availability of a complete genome assembly for the *C. parvum* IOWA II strain will facilitate studies aimed at understanding the biology, pathogenesis, and transmission of this important pathogen. It will also provide a valuable resource for the development of new diagnostics, drugs, and vaccines against cryptosporidiosis.

## Methods

### Oocyst material

Oocysts for *Cp*BGF were obtained from a commercial source (Bunch Grass Farms, Deary, ID, USA) in 2017. A total of 10^8^ oocysts were incubated on ice for 10 min in household bleach (diluted 1: 4 in water) and then washed twice with cold phosphate-buffered saline (PBS). Excystation of sporozoites was then induced by incubating oocysts in 0.8% sodium taurodeoxycholate (Sigma) in PBS at 37 °C for 1 h. To maintain the potential for high molecular weight (HMW) for long read sequencing, sporozoite genomic DNA was extracted using the traditional phenol-chloroform DNA extraction method^14^.

### Whole genome sequencing and assembly

PE-300 reads were generated on a MiSeq Illumina platform. Reads were quality-checked and trimmed using Trimmomatic v0.39^15^. ONT library preparation was performed using the SQK-LSK109 Ligation Sequencing Kit and the Rapid Barcoding Sequencing Kit (Oxford Nanopore Technologies, Oxford, UK) following the manufacturer’s instructions. The library was sequenced on an ONT MinION device with R9.4.1 flow cells. Genome assembly was performed using two long-read assembly approaches: (i) Flye v2.8.2^16^; and (ii) NECAT v0.3.3^17^. Both assemblies were evaluated, and the structures were compared to identify any regions that differed. NextPolish v2.3.0^18^ was used to correct base call errors in the assembly using the *Cp*BGF short-read sequencing data. The NECAT genome was chosen as the reference model since it was able to better resolve the telomeric regions, and Geneious prime (v2019) was used to manually curate regions that needed correction. Those structural corrections were all supported by long read alignments generated using Minimap2 v2.24^19^. Genome statistics were calculated using QUAST v5.0.2^20^. Telomeres were detected using the python script FindTelomeres (https://github.com/JanaSperschneider/FindTelomeres) looking for patterns at the start and end of the contigs only. The sequence of the *Cryptosporidium* telomere repeat is “CCTAAA/ AGGTTT”, but the detection also works with the default settings available in the original script. Benchmarking Universal Single-Copy Orthology (BUSCO) software v5.4.6^21^ was used to check for completeness of the assembly using the Apicomplexa database (https://busco-data.ezlab.org/v5/data/lineages/apicomplexa_odb10.2024-01-08.tar.gz).

### RNA sequencing

4.0 × 10^8^ excysted *Cp*BGF sporozoites were incubated with 1 ml of RLT buffer and 10 μl of β-mercaptoethanol (Sigma-Aldrich). RNA was extracted using the “Animal Cells” protocol of the RNeasy Mini Kit (QIAGEN). Extracted RNA was treated with DNase I (Invitrogen). 1 μg of total RNA was used for PacBio Iso-Seq library construction and the library was sequenced on a PacBio Sequel II system at the Georgia Genomics and Bioinformatics Core.

### Genome annotation

Genome annotation was performed: (i) by transferring gene annotations based on homology using Liftoff v1.6.3^22^ using the *C. parvum* IOWA-ATCC genome as a reference, and (ii) by generating an *ab initio* prediction with BRAKER2^23^ trained using a mix of available *Cp*BGF RNAseq data as was done previously for *Cp*IOWA-ATCC^10^. Both annotation tracks were added to a WebApollo2 environment^24^ for manual curation. For additional new lncRNA gene annotation, we used TAMA^25^ combined to Iso-seq data. For lncRNA gene models, genes must be: (i) over 200 bp long; and (ii) have a predicted CDS smaller than 200 bp (90 amino acids) to be annotated as lncRNA.

### Gene naming

To facilitate the translation of gene IDs between the CpBGF genome and the original reference *Cp*IOWA II, we developed a gene ID nomenclature similar to the original reference nomenclature, structured as “(locustag)_(chr#)00(Genenumber)” due to changes in GenBank gene ID naming rules. For instance, the *gp60* gene in the reference genome sequence is labeled as cgd6_1080, while in our annotation, it is designated as cpbgf_6001080. Most genes retained their original gene numbers in agreement with their orthologs, verified using Orthofinder^26^. In cases where paralogs were identified, manual curation was conducted via WebApollo2 to ensure proper naming. Exceptions included genes that have had their annotation fused or split, supported by long-read transcriptomic data, as well as genes relocated to different chromosomes in this new assembly— *e.g*., reference gene cdg4_380 is located on a different chromosome in our long-read assembly, it has gene ID, CpBGF_6004926 and cgd5_4510 is now found on Chromosomes 1, 7 and 8 (the replicated chromosome ends). When a gene is detected on a new chromosome, it will receive a new geneID consistent with its new chromosomal location. 44 such genes were identified. If it is located in a non-syntenic location on the same chromosome, it will keep its existing geneID and will appear out of sequence numerically. For syntenic genes, gene numbering is based on intervals of 10. When incorporating ncRNA information (including tRNAs, rRNAs, sncRNAs, and lncRNAs) or newly identified genes from expression data or splits of existing genes, the numbering was adjusted according to the features and their positions relative to neighboring annotated genes. For example, a gene annotated between two others typically ends in a 5, while newly added genes were assigned odd numbers (3, 5, and 7), with 3 and 7 closer to their upstream and downstream neighbors.

### Gene structure and UTR length curation using iso-seq

Two major issues for *Cryptosporidium* genome annotation are the detection of untranslated region (UTR) boundaries and the validation of annotated gene structures. Compactness and antisense transcription cause frequent UTR overlaps, which makes it difficult to differentiate gene boundaries using short-read RNAseq data. To solve this problem and generate more reliable UTR annotation predictions, we utilized sporozoite total RNA sequenced using PacBio Iso-Seq. Iso-Seq data were processed using SMRT LINK v11.0 (PacBio, CA) and the Transcriptome Annotation by Modular Algorithms, TAMA^25^. First, raw reads were quality-filtered to remove sequences with either incomplete read cycles or lacking a poly-A tail. Filtered circular consensus sequencing (CCS) reads are subsequently trimmed of adapters and poly-A tails, then collapsed into linear full-length long reads with PacBio’s SMRTlink tools. Reads are mapped to the *Cp*BGF genome using minimap2 v.2.24^19^. The mapped reads are then fed into the TAMA pipeline to be collapsed into transcript models using TAMA’s high stringency mode. ORFs were predicted and assigned to individual transcript models using NCBI BLAST. The TAMA pipeline-generated transcript models were used in conjunction with transcript models obtained from the annotation pipeline to update the gene model structures, including UTRs.

The UTR boundaries were determined collectively using both Iso-Seq and 21 publicly available stranded short-read RNAseq data sets obtained from NCBI SRAs in bioproject PRJNA530692 (SRR8841041 - SRR8841061)^27^ and 17 NCBI SRAs in bioproject PRJEB25665^28^ (ERR2433389 - ERR2433405). A UTR boundary cutoff requiring >90% read consensus for specific transcription start and end sites was used. These approaches helped generate full-length transcripts for expressed genes, facilitating the genome annotation and determination of UTR boundaries. These tracks were also added to a private Web Apollo 2 instance for use in manual curation across the entire *Cp*BGF assembly.

### Gene function annotation

Gene function annotation was performed by comparing the predicted protein sequences to previous predictions made for *Cp*IOWA-ATCC^10^, which was supported by protein sequence similarity using BLAST against Swiss-Prot, TrEMBL, and the NCBI nonredundant protein database, I-TASSER v5.1^29^ and Interproscan v5^30^. The best hit with an E-value cutoff of 1E-5 or better was used to assign putative functions to the genes. This approach benefits from the previous annotation and updates generated by the *Cryptosporidium* community and submitted to repositories. Existing genes with no functional information available from our approach kept their functional annotation as originally described in *Cp*IOWA II if present.

Non-coding RNA annotation was performed using tRNA-scan-SE v2.0.7^31^ and Infernal v1.1.3^32^ which can detect most of the known ncRNA families such as tRNAs and rRNAs. Additionally, *Cryptosporidium* long non-coding and small non-coding RNAs described by Li *et al*.^12,13^ were added.

### Comparison with previous *C. parvum* assemblies

Structural comparative analysis was performed using Minimap2 v2.24^19^, Samtools v.1.16.1^33^ and D-GENIES v1.5.0^34^ to identify syntenic regions among the available chromosomal level assemblies. Dot plot analysis is done through D-GENIES web server using default minimap2 parameters. Gene orthology was determined using OrthoFinder v2.5.4^26^ to identify orthologous genes and detect potential duplication events. The orthology information was also useful to generate gene IDs similar to the original *Cp*IOWA II genome annotation. Using similar ID numbers will benefit the community.

To compare single nucleotide variants (SNVs) across the available isolates, we used the Genome Analysis Toolkit v.4.1.4.0 (GATK)^35^. This software was chosen for its ability to detect small variants such as SNPs and indels with high sensitivity and specificity. We first aligned the reads from different available *C. parvum* IOWA isolates to the new *Cp*BGF genome sequence using the BWA-MEM v0.7.12^36^ alignment tool and then GATK to call variants across all samples. All called variants were then submitted to a variant annotation pipeline using snpEFF v5.1^37^. The results from this analysis are used to assess the suitability of the new genome sequence for the community with regards to variability across isolates. The GP60 protein sequence alignment was performed using the Clustal Omega v1.2.4 web server (https://www.ebi.ac.uk/jdispatcher/msa/clustalo).

## Data Records

The raw data associated with this work are available at the NCBI Sequence Read Archive (refs. ^38–40^). The assembly and annotation are available at GenBank (41), as depicted in Table 1. All relevant information regarding the sequencing methods and analysis pipeline are provided to ensure the reproducibility of the results.

**Table 1:** Sequence data generated for the haploid *Cryptosporidium parvum* IOWA II-BGF isolate.

Data type	Technologies Utilized	Year	Accession number and Reference
Hybrid Genome Assembly	Oxford Nanopore, MiSeq	2024	ASM3523276v1/GCA_035232765.1^44^
Long DNA reads	Oxford Nanopore	2019	SRP307938^38^
Short DNA reads	Illumina MiSeq	2020	*SRP255839^39^
RNA single-molecule Iso-Seq	PacBio Sequel II	2024	SRP504065^40^

*Data generated at UGA but submitted from UPENN following move of the Striepen Lab. Note: Some of the accessions above are associated with two different over-arching BioProjects (PRJNA983265 and PRJNA573722), that include additional *Cp* isolates, but the accessions here are *Cp*BGF.

## Technical Validation

### Parasite strain

**Fig. 1:**
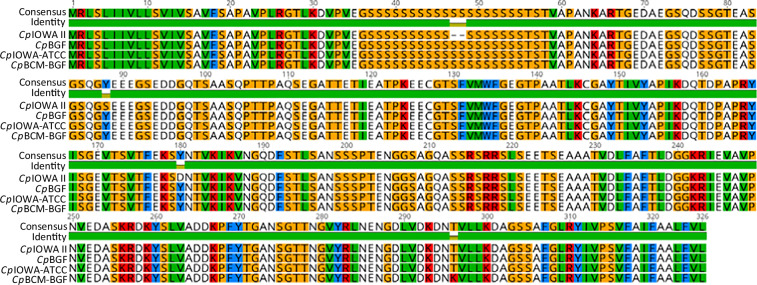
Amino acid alignment between GP60 sequences from all four *C. parvum* IOWA II genome assemblies analyzed.

All genome sequences were checked for their encoded GP60 type to confirm that they were type IIa according to the community strain typing standard^41^. Due to the lack of genome annotation for *Cp*BCM-BGF assembly, we utilized *Cp*BGF’s *gp60* mRNA as a query to obtain the *Cp*BCM-BGF *gp60* sequence via the BLASTn algorithm with default parameters. *Cp*BCM-BGF *gp60* was subsequently translated to protein. We note that small differences are observed between the *C. parvum* IOWA II isolates compared in this study. The differences observed between the *Cp*IOWA II sequence and the others may be caused by accumulated differences over time and their different passage histories. *Cp*BCM-BGF also shows an inferred amino acid difference at position 296 (Fig. 1).

### How the new genome assembly and annotation compare to previous data sets

Analysis revealed that the *Cp*BGF genome sequence is the first telomere-to-telomere genome assembly for *C. parvum*. Other than the genome size, the major structural difference between *Cp*BGF and the other publicly available long-read generated genome assemblies e.g., *Cp*IOWA-ATCC^10^ and *Cp*BCM-BGF^11^, is the completeness of the subtelomeric and telomere regions (Table 2).

**Table 2:** Assembly statistics of available *C. parvum* IOWA II genome sequences.

Statistics	CpIOWA II^a^	CpIOWA-ATCC^b^	CpBCM-BGF^c^	CpBGF
# of contigs	8	8	8	8
Total length	9,102,324	9,122,263	9,197,619	9,259,183
Largest contig	1,344,712	1,332,634	1,355,650	1,379,419
N50	1,104,417	1,108,396	1,108,772	1,107,426
L50	4	4	4	4
GC%	30.22	30.18	30.11	30.04
N’s	18,558	0	0	0
# of assembled telomeres	3	13	12	16

^a^(Abrahamsen *et al*.)^8^; ^b^(Baptista *et al*.)^10^; ^c^(Menon *et al*.)^11^.

**Fig. 2:**
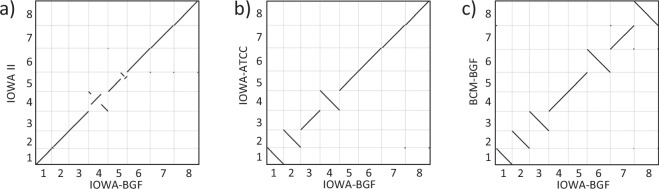
Genome comparison between the available chromosomal level *C. parvum* assemblies. (**a**) Dot plot alignment of *Cp*BGF compared to the *Cp*IOWA II showing the translocations observed on chromosomes 2, 4 and 5. (**b,c**) Dot plot comparison of *Cp*BGF to *Cp*IOWA-ATCC and *Cp*BCM-BGF showing good correlation but differences in chromosomal orientation.

When comparing all four *C. parvum* IOWA II genome sequence assemblies for synteny, we can observe that the *Cp*BGF genome sequence has been oriented to follow the same chromosome level orientation as the original genome assembly, CpIOWA II, (Fig. 2).

### Genome annotation

The hybrid genome assembly was annotated using the *Cp*IOWA-ATCC genome annotation^10^, complemented with annotated lncRNA^12^ and sncRNA genes^13^, new data available from long-read Pacbio RNA Iso-Seq, and publically available strand-specific short-read RNAseq obtained from NCBI (See Data Records), Table 3. New lncRNA gene annotations were generated for novel Iso-Seq TAMA gene models that were >200 bp and had a predicted CDS of <200 bp (90 amino acids). The *Cp*BGF genome annotation uses similar gene IDs based on syntenic, homologous genes in the *Cp*IOWA II reference annotation, which facilitates linkage to published gene IDs. For example, the commonly used *gp60* gene, reference gene ID cgd6_1080 is cpbgf_6001080 where the first digit after the underscore signifies the chromosome number. Please see the methods for greater detail and a link to a geneID mapping table.

**Table 3:** Annotation statistics of available *C. parvum* IOWA II genome sequences.

Annotation Statistics	*Cp*IOWA II 2004^a^	*Cp*IOWA II 2018^b^	*Cp*IOWA-ATCC 2022^c^	*Cp*BGF
# Protein coding genes	3805	3941	3892	3925
# mRNA including isoforms	3805	3941	3896	3929
# lncRNA genes	0	0	400	766
# sncRNA genes*	20	29	76	87
# tRNA genes	45	45	45	45
# rRNA genes	15	15	15	15
Annotation contributing to transcripts (bp)	6,858,273	7,430,054	7,248,326	8,204,860
Genome % contributing to annotated genes	75.34%	81.6%	79.45%	88.61%

^a^Data record obtained through CryptoDB release 24 (Abrahamsen *et al*.)^8^. ^b^2018 re-annotation with short-read RNAseq annotated UTRs. Data record obtained through CryptoDB release 37. ^c^New genome assembly and annotation including lncRNA and sncRNAs but not UTRs. Data record obtained from CryptoDB release 60, (Baptista *et al*.)^10^. lncRNA - long noncoding RNA. sncRNA – small noncoding RNA. *sncRNAs include snRNA, snoRNA and SRP-RNA.

**Fig. 3:**
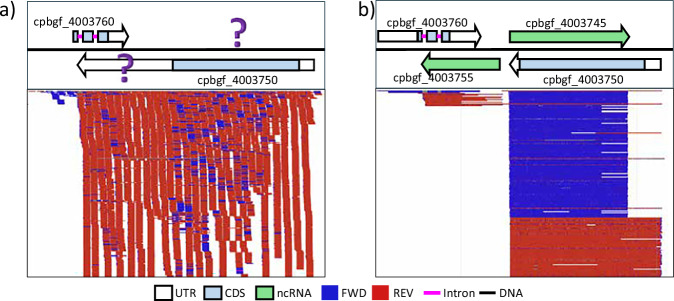
Comparative annotation of same genome region with short-read versus long-read RNAseq. (**a**) Protein coding genes annotated with short-read RNAseq data only. Question marks indicate ambiguous annotation of untranslated region (UTR) coordinates due to overlapping transcripts. (**b**) Long-read RNAseq updated gene models with resolved UTRs for the exact same protein-coding genes. Novel antisense noncoding RNA genes (green) are annotated with read support.

As evidenced in Fig. 3a, traditional short-read data are unable to resolve the ambiguity of gene boundaries due to overlapping mapped reads from different strands that span multiple gene models. To address this challenge, we used single-molecule long-read RNAseq that captures full-length reads. Figure 3b, shows resolution for the same region as in Fig. 3a, including UTRs for the two protein-coding genes and annotation of two new ncRNA genes.

### Differences in single-nucleotide variants between *C. parvum* IOWA II genome assemblies

**Fig. 4:**
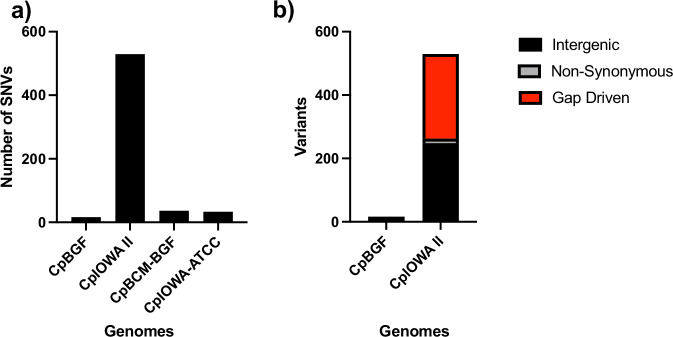
Comparative SNV distribution across all four genome sequences. (**a**) total number of variants found when compared to *Cp*BGF. (**b**) Variant types found in *Cp*BGF and *Cp*IOWA II separated by genome feature location.

Variant analysis revealed a high degree of conservation among the four genome sequence assemblies with only a few variants detected when compared to *Cp*BGF Illumina short reads (Fig. 4a). A total of 16 variants in 12 *Cp*IOWA II genes were found to be non-synonymous (missense_variants, cgd1_750, cgd1_1840, cgd3_3370, cgd5_130, cgd7_3030, cgd7_4990, cgd7_5040; frameshift_variants cgd1_750, cgd2_3680, cgd3_1180 (2 frameshifts), cgd3_3630; stop codon gained cgd4_1380; frameshift_variant and stop codon lost cgd8_1270). Most variants are intergenic or reside in gaps (Fig. 4b).

**Fig. 5:**
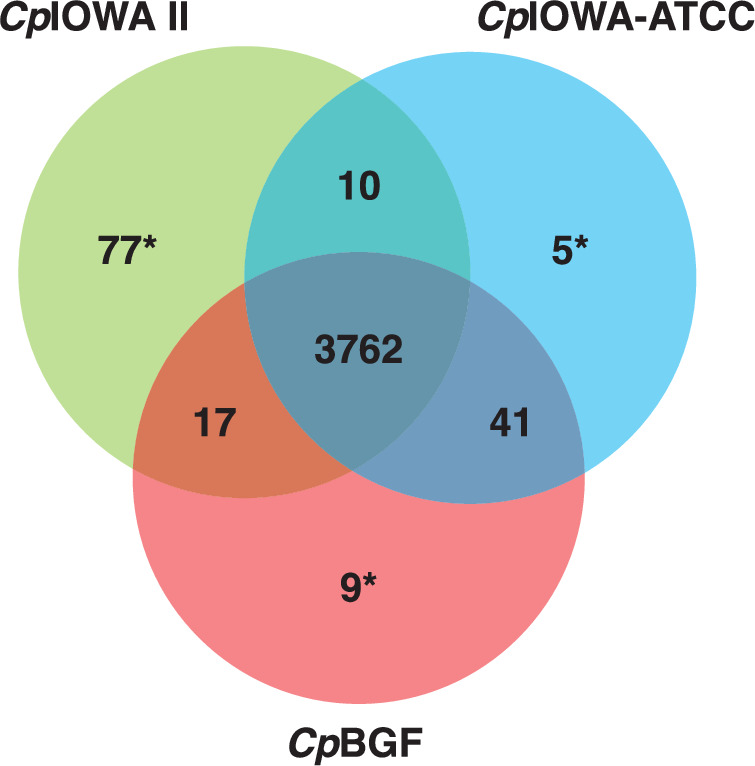
Venn diagram indicating the number of shared Orthologue groups. Singletons observed between *Cp*BCM-BGF and *Cp*BGF are related to assembly limitations, such as missing subtelomeric regions in *Cp*BCM-BGF. *Singletons – Proteins with no shared ortholog detected in annotated proteins.

Orthology analysis was performed using OrthoFinder to identify genes shared by the four isolates as currently annotated (Fig. 5). *Cp*IOWA II has the largest number of singletons due to sequences not matching the updated annotated genes and missing regions (i.e. truncated genes) spanning assembly gaps. The differences between *Cp*IOWA-ATCC and *Cp*BGF are related to the subtelomeric regions that were limited for the *Cp*IOWA-ATCC annotation and CpIOWA II reference assembly and reannotation. Overall, the orthology analysis provided insights into the assembly completeness and the genetic relatedness of the *C. parvum* IOWA isolate samples.

To validate the quality of the *Cryptosporidium parvum* IOWA-BGF genome assembly, we used BUSCO to search apicomplexan databases containing a total of 446 orthologous single-copy genes. The results showed that 96.2% of the complete single-copy genes were retrieved, of which none were duplicated. Only 0.9% of BUSCO genes were identified as fragmented, and 3.1% were missing from the genome. These results indicate that the genome assembly has high completeness.

To further validate the accuracy of the genome assembly, we aligned the filtered short Illumina reads back to the genome assembly using BBmap v.38.93 software^42^. Approximately 95% of the short reads were mapped to the genome. The ratios of multiallelic single nucleotide polymorphisms (SNPs) were 0%, indicating that the assembly had high single, base-level accuracy. Overall, the high BUSCO completeness scores and accurate mapping of Illumina reads to the long-read assembly support the quality and reliability of the new *Cp*BGF genome assembly.

**Fig. 6:**
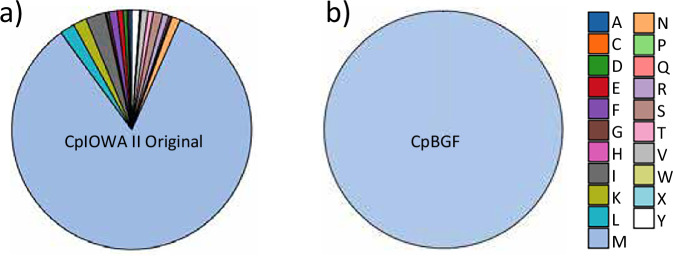
Composition of starting amino acid for all protein coding genes. (**a**) *Cp*IOWA II from CryptoDB release 24, 3805 total protein coding genes. (**b**) *Cp*BGF with 3923 total protein coding genes.

We validated whether the predicted proteins, including isoforms, start with a Methionine (M). We show that all *Cp*BGF predicted proteins have a methionine as the first amino acid, Fig. 6. In comparison, 636 predicted reference *Cp*IOWA II proteins start with non-methionine amino acids (Fig. 6). All *Cp*IOWA-ATCC predicted proteins^10^ begin with a methionine (data not shown). The average predicted protein sizes are similar among annotations for the three genome assemblies, with *Cp*IOWA II at 597 aa, *Cp*IOWA-ATCC at 599 aa, and *Cp*BGF at 596 aa.

## Usage Notes

### Prior historical *Cryptosporidium* genome data records used for comparisons

The reference *C. parvum* IOWA II whole genome assembly and annotation was published in 2004^43^ (GenBank assembly GCA_000165345.1). The annotation was updated in 2018 and first appeared in CryptoDB.org release 37. A more complete *C. parvum* IOWA II (DNA obtained from ATCC) genome assembly and new annotation with all but three contiguous telomeres and adjacent genes was published in 2022^10^ (GCA_015245375.1 and appeared in CryptoDB.org release 52), the predicted remaining 3 chromosome ends including subtelomeric regions were deposited in the GenBank as nucleotide sequences under the accessions MZ892386, MZ892387 and MZ892388. The *Cp*IOWA-ATCC assembly did not have all chromosomes oriented as in the 2004 IOWA II reference assembly and utilized its own gene naming system. The *Cp*IOWA-ATCC assembly had annotation updates in 2022 and 2023 (CryptoDB.org releases 59 and 63) to include the addition of lncRNAs. In 2022, a new unannotated assembly for C. *parvum* IOWA II with 12 assembled telomeres (named here as CpBCM-BGF) was submitted to GenBank (GCA_019844115.2) by the Baylor College of Medicine^11^. Here we present a full T2T assembly and annotation for *C. parvum* IOWA II (GCA_035232765.1) named *Cp*BGF. For complex reasons related to overlapping gene features, the ncRNAs contained in the GenBank annotation appear and disappear from the GenBank record. We are working on a solution with GenBank. The GFF and fasta files corresponding to the *Cp*BGF annotation and assembly presented here (CpBGF_genome_V1) and all prior CryptoDB.org versions mentioned are available from (https://github.com/jkissing/CpBGF_Repository). A table containing the geneID mapping from *Cp*BGF to *C. parvum* IOWA II is also provided in this repository. The table is quite useful for understanding genes that do not have a one-to-one mapping conversion.

## Data Availability

All code and commands used in this project are presented below and in a public github repository (https://github.com/jkissing/CpBGF_Repository): • ONT gDNA basecalling: • Dorado v0.5.0: dorado basecaller \ -r --emit-fastq \ dna_r9.4.1_e8_sup@v3.6 \< input_fast5 >  > CpBGF_ONT_raw.fastq • Chimeric read detection: • Minimap2/yacrd: minimap2 -x ava-ont -g 500 CpBGF_ONT_raw.fastq CpBGF_ONT_raw.fastq > overlap.paf yacrd -i overlap.paf -o reads.yacrd • Genome Assembly: • NECAT/0.0.1: necat.pl correct necat_config.txt necat.pl assemble necat_config.txt necat.pl bridge necat_config.txt • necat_config.txt: PROJECT = BGF_NECAT ONT_READ_LIST = BGF_ONT.txt GENOME_SIZE = 9200000 THREADS = 5 MIN_READ_LENGTH = 3000 PREP_OUTPUT_COVERAGE = 30 OVLP_FAST_OPTIONS = -n 500 -z 20 -b 2000 -e 0.5 -j 0 -u 1 -a 1000 OVLP_SENSITIVE_OPTIONS = -n 500 -z 10 -e 0.5 -j 0 -u 1 -a 1000 CNS_FAST_OPTIONS = -a 2000 -x 4 -y 12 -l 1000 -e 0.5 -p 0.8 -u 0 CNS_SENSITIVE_OPTIONS = -a 2000 -x 4 -y 12 -l 1000 -e 0.5 -p 0.8 -u 0 TRIM_OVLP_OPTIONS = -n 100 -z 10 -b 2000 -e 0.5 -j 1 -u 1 -a 400 ASM_OVLP_OPTIONS = -n 100 -z 10 -b 2000 -e 0.5 -j 1 -u 0 -a 400 NUM_ITER = 2 CNS_OUTPUT_COVERAGE = 30 CLEANUP = 1 USE_GRID = false GRID_NODE = 0 SMALL_MEMORY = 0 POLISH_CONTIGS = true • Genome Polishing: NextPolish v.1.2.4: sgs_options = -max_depth 200, lgs_options = -min_read_len 1k -max_read_len 100k, andlgs_minimap2_options = -x map-ont. • Genome Annotation: • BRAKER2: braker.pl--species = yourSpecies--genome = genome.fasta \ --rnaseq_sets_ids = SRA_ID1,SRA_ID2 \ --rnaseq_sets_dirs = /path/to/local/fastq/files/ \ --UTR = on--addUTR = on--ab_initio • Liftoff: liftoff -g *Cp*IOWA-ATCC_reference.gff -o CpBGF_liftoff.gff -copies -cdsCpBGF_genome.fasta *Cp*IOWA-ATCC_genome.fasta • Calculation for Annotation contributing to transcripts (bp): awk ‘$1!~ /^#/ & & $3! = “region” {print $1”\t”$4”\t”$5}’ INPUT.gff | \ bedtools sort | bedtools merge | \ awk ‘{SUM +  = ($3 - $2 + 1)} END {print SUM}’ • Iso-Seq analysis: • SMRTlink v10.1: isoseq. 3 refine \ Isoseq3_ccs.fl.NEB_5p--NEB_Clontech_3p.bam \ primers.fasta cp.flnc.bam \ -j 8--require-polya -v pbmm2 index cp_bgf.fa cp_bgf.mmi pbmm2 align \ cp_bgf.mmi cp.flnc.bam cp_bgf_isoseq_aligned.bam \ --sort -j 8 -J 8--preset ISOSEQ \ -G 2500--log-level INFO • TAMA: tama_collapse.py -s tama/ cp_bgf_isoseq_aligned.sam \ -f cp_bgf.fa -p tama_cp_bgf \ -x no_cap -d merge_dup \ -a 100 -z 100 \ -sj sj_priority -lde 1 -sjt 20 • BUSCO run: busco -i < genome.fasta > -l./apicomplexa_odb10 -m genome -o < output > • Short-read RNAseq mapping: •STAR v2.7.10: STAR--runMode alignReads \ --runThreadN 16 \ --genomeDir STAR_genome \ --readFilesIn < input_read_1.fq >  < input_read_2.fq > \ --outSAMtype BAM SortedByCoordinate \ --quantMode GeneCounts \ --bamRemoveDuplicatesType UniqueIdentical \ --twopassMode Basic \ --outFileNamePrefix < output_name > \ --outWigStrand Stranded \ --outSAMstrandField intronMotif \ --alignIntronMax 2500 \ --chimOutType Junctions \ --limitBAMsortRAM 1000000000 • Protein sequence extraction: • AGAT v0.9.2: agat_sp_extract_sequences.pl -p \ -g < input.gff > -f < genome.fa > \ -o < protein_output.fa > • Starting amino acid analysis: • Seqkit v2.5.1: seqkit fx2tab < protein_input.fa > \ | cut -f 2 | awk ‘{print substr($1,1,1)}’ \ | sort -V | uniq -c >  < output.txt > • Variant Call: • BWA/SAMTOOLS: bwa mem -v 2 -M -t 10 $GENOME $R1 $R2 > $SAMPLE.sam samtools view -b -S -o $SAMPLE.bam $SAMPLE.sam • PICARD: java -Xmx2g -classpath “$PICARD” -jar $SortSam INPUT = $SAMPLE.bamOUTPUT = $SAMPLE.s.bam VALIDATION_STRINGENCY = LENIENT SORT_ORDER = coordinate java -Xmx2g -classpath “$PICARD” -jar $MarkDuplicates INPUT = $SAMPLE.s.bamOUTPUT = $SAMPLE.sd.bam METRICS_FILE = $SAMPLE.dedup.metricsREMOVE_DUPLICATES = false VALIDATION_STRINGENCY = LENIENTASSUME_SORTED = true java -Xmx2g -classpath “$PICARD” -jar $ARreadgroups INPUT = $SAMPLE.sd.bamOUTPUT = $SAMPLE.sdr.bam SORT_ORDER = coordinate RGID = $SAMPLE RGLB = $SAMPLERGPL = illumina RGPU = $SAMPLE RGSM = $SAMPLE VALIDATION_STRINGENCY = LENIENT java -Xmx2g -classpath “$PICARD” -jar $BuildBamIndex INPUT = $SAMPLE.sdr.bam VALIDATION_STRINGENCY = LENIENT • GATK: gatk HaplotypeCaller -R $GENOME -I $SAMPLE.sdrsm.bam -O $SAMPLE.GATK.vcf -ploidy$PLOIDY -stand-call-conf 30 gatk VariantFiltration -R $GENOME -V $SAMPLE.GATK.vcf -O $SAMPLE.GATK_Filtered.vcf-cluster 3 -window 10 -filter “QUAL < 30.0 || DP < 10 || QD < 1.5 || MQ < 25.0”--filter-name “StdFilter” -filter “MQ0 >  = 4 & & ((MQ0/(1.0 * DP)) > 0.1)”--filter-name“HARD_TO_VALIDATE” -filter “MQ < 40.0 || FS > 60.0”--filter-name “gatkFilter” Other commands and pipelines used in data processing were executed using their corresponding default parameters. Bedtools is used for calculating the number of base pairs contributing to annotations through merging genomic regions with overlapping gene features.
